# Role of cognitive reserve in progression from mild cognitive impairment to dementia

**DOI:** 10.1590/S1980-57642010DN40100005

**Published:** 2010

**Authors:** Ricardo F. Allegri, Fernando E. Taragano, Hugo Krupitzki, Cecilia M. Serrano, Carol Dillon, Diego Sarasola, Mónica Feldman, Graciela Tufró, María Martelli, Viviana Sanchez

**Affiliations:** Servicio de Neuropsicología (SIREN) y Unidad de Investigación “Rene Barón” del Instituto Universitario CEMIC, Buenos Aires, Argentina.

**Keywords:** mild cognitive impairment, risk factors, dementia, cognitive reserve

## Abstract

Cognitive reserve is the ability to optimize performance through differential recruitment of brain networks, which may reflect the use of alternative cognitive strategies.

**Objectives:**

To identify factors related to cognitive reserve associated with progression from mild cognitive impairment (MCI) to degenerative dementia.

**Methods:**

A cohort of 239 subjects with MCI (age: 72.2±8.1 years, 58% women, education: 12 years) was assessed and followed for five years (2001 to 2006).

**Results:**

In the first year, 13.7% of MCI converted to dementia and 34.7% converted within three years (78.3% converted to Alzheimer’s dementia). Risk factors for those who converted were education less than 12 years, MMSE score less than 27, Boston naming test score less than 51, IQ (Intelligence Quotient) less than 111, age over 75 years, lack of occupation at retirement, and presence of intrusions in memory recall (all account for 56% of the variability of conversion).

**Conclusions:**

MCI patients are a population at high risk for dementia. The study of risk factors (e.g. IQ, education and occupation), particularly those related to cognitive reserve, can contribute important evidence to guide the decision-making process in routine clinical activity and public health policy.

## Abstract (pt)

Reserva cognitiva é a habilidade em otimizar o desempenho através do recrutamento de redes neurais, que talvez reflitam o uso de estratégias cognitivas alternativas.

**Objetivos:**

Identificar fatores relacionados à reserva cognitiva associados à progressão do comprometimento cognitivo leve (CCL) para demência degenerativa.

**Métodos:**

Uma coorte de 239 indivíduos com CCL (idade: 72.2±8.1anos, 58% mulheres, educação: 12 anos) foram avaliados e seguidos por cinco anos (2001-2006).

**Resultados:**

No primeiro ano 13.7% dos CCL converteram para demência e 34.7% em três anos (78.3% converteram para doença de Alzheimer). Os fatores de risco para aqueles que converteram foram: educação menor do que 12 anos, MMSE menor do que 27, teste de Nomeação de Boston menor do que 51, QI (Quociente de Inteligência) menor do que 111, idade superior a 75 anos, falta de ocupação na aposentadoria, e presença de intrusões na memória de evocação (todos contando para 56% da variabilidade de conversão).

**Conclusões:**

Pacientes com CCL são uma população de risco para demência. O estudo dos fatores de risco (como QI, educação e ocupação), principalmente, aqueles relacionados à reserva cognitiva podem contribuir para uma evidência importante para o processo de decisões na atividade clínica e na saúde pública.

Cognitive reserve is the ability to optimize performance through differential recruitment of brain networks, which may reflect the use of alternative cognitive strategies. The idea of reserve against brain damage stems from the repeated observation that there does not appear to be a direct relationship between the degree of brain pathology and the clinical manifestation of the damage.^1^ Several studies have suggested that differential susceptibility to dementia level is related to variables such as education, literacy, IQ and engagement in leisure activities.^2-7^ The concept of cognitive reserve posits that individual differences in how tasks are processed might provide differential reserve against brain pathology.^8^

Although cognitive decline without dementia has commonly been considered a normal consequence of brain aging, cognitive impairment can mark the onset of dementia. A number of clinical definitions have been proposed to describe these cognitive deficits. Mild cognitive impairment (MCI) was defined by Petersen et al.^9^ as a transitional state that can precede dementia; however, conversion rates remain controversial.

The development of cognitive reserve is associated with genetic predisposition and exposure to and interaction with favorable environments (education, engagement in cognitively stimulating activities and occupation).^7^ However, limited data are available regarding the role of cognitive reserve in conversion from MCI to dementia.^2-5^

This investigation analyzed the conversion from MCI to dementia in our “CEMIC cohort”, and explored the risk factors related to cognitive reserve associated with transition in patients at risk of dementia.

## Methods

### Design and setting

This was a prospective cohort study of outpatients with MCI (CEMIC Cohort). The study was performed with the approval of the institutional review board. Each participant or his/her legal representative provided informed consent for participation.

### Subjects

Between January 2001 and January 2006, 1491 consecutive outpatients were screened at our Dementia Clinic (Servicio de Investigación Neuropsicológica, SIREN) at the CEMIC Institute. Of these subjects, 239 met inclusion criteria for mild cognitive impairment^10^ and were followed at least twice every 4 months. Patients were referred by general practitioners (45%), neurologists (27%), psychiatrists (16%) and others (12%). Subjects were typically referred because they had experienced cognitive impairment at work or in activities of daily living, or because they were worried about their cognitive functioning.

### Procedures

Data collected at baseline included socio-demographic and clinical variables including age, education level expressed in years, gender, marital status, retirement status, occupational status, socio-economic level and number of consultations. Each subject underwent a uniform structured evaluation, including medical history, complete neurological examination; neuropsychological assessment (see below) and the Beck Depression Inventory.^11^ Physical examination and laboratory tests were performed as clinically appropriate for each patient. Neuro-imaging examinations using brain CT scan, MRI or SPECT, as appropriate, were assessed.

#### Neuropsychological assessment

At baseline, patients were assessed with an extensive neuropsychological battery that included the Mini Mental State Examination, MMSE^12^ (a validated^14^ Argentine adaptation^13^), Signoret Memory Battery,^15^ Boston Naming Test^16^ (local Spanish adaptation^17^), Verbal Fluency,^18^ Trail making test^19^ and Wechsler Abbreviated Scale of Intelligence- WASI.^20^

#### Clinical diagnosis

Dementia diagnoses were made according to the Diagnostic and Statistical Manual of Mental Disorders, Fourth Edition^21^ while AD diagnoses were based on the National Institute of Neurological and Communicative Disorders and Stroke-Alzheimer's Disease and related Disorders of Association criteria, respectively.^22^

A diagnosis of MCI was reached if the patient met the following criteria:^10^

1. The individual was neither normal nor demented;
2. There was evidence of cognitive impairment, shown by either objectively measured decline over time or subjective report of decline by self or informant in conjunction with objective cognitive deficits;
3. Activities of daily living were preserved and complex instrumental functions were either intact or minimally impaired. In this study, we considered evidence of cognitive deficit as when one of the objective neuropsychological tests showed at least 1.5 SD below the mean value for age- and education-matched healthy subjects.

Patients were excluded from the cohort if they had cerebrovascular disorders (defined by a score of 5 or higher on the Hachinski Ischemic Score)^23^ or a history of neurological or major psychiatric disease or unstable general medical conditions.

Raters examined each patient and both of the senior examiners (FT, RFA) reviewed data from each visit to determine the diagnosis of MCI at each time point and to ascertain whether a given patient had converted to dementia.

#### Follow up and outcome assessment

Patients were assessed at baseline and every 4 months or when necessary, using a comprehensive approach. Longitudinal analyses were based on completers with more than 2 evaluations. The median follow up for MCI patients was 24 months.

### Statistical analysis

Categorical variables were expressed as percentages and for continuous variables, mean and standard deviations were estimated, while for non-normally distributed variables, medians and percentiles were considered. To compare frequency differences by diagnosis of conversion or non-conversion to dementia, univariate analyses were performed using the Chi-square test. Student t-tests were used to compare continuous variables between groups, while the nonparametric Wilcoxon rank sum test was applied to compare non-normally distributed variables between groups. Survival analyses were then performed to assess the association between time to onset of dementia and the analyzed variables. The main outcome was a diagnosis of dementia. Time to this event was considered an outcome of interest. The follow-up period was from the initial observation to conversion to dementia or to the study end point. Cox proportional hazards models were also estimated to test the multivariate associations between multiple explanatory variables and conversion to dementia in patients with MCI. Effects are shown as hazard ratios (HR), with 95% confidence intervals (95%CI). For all analyses, the STATA 8.0 statistical software package was used.

## Results

239 participants were followed up for 5 years (median 24.15 months; 10^th^ percentile: 9 and 90^th^ percentile: 51.8). Demographic data are shown in Table 1. Loss during follow-up (including death) was less than 12%.

**Table 1:** Demographic data.

Patients (number)	239	
Age at entry (mean±SD)	72.3±7.8	
Sex (male, number and %)	98	41%
Marital Status (Married, number and %)	159	67%
Education (median in years)	12	
Work (retired, number and %)	182	76%
Follow-up		
Median (months)	24.15	
10^th^ Percentile	9.00	
90^th^ Percentile	51.89	
Number of visits		
Median	7	
10^th^ Percentile	2	
90^th^ Percentile	21	
MMSE score (median)	28	
CDR score (median)	0.5	

### Conversion to dementia in patients with MCI

**Figure 1:**
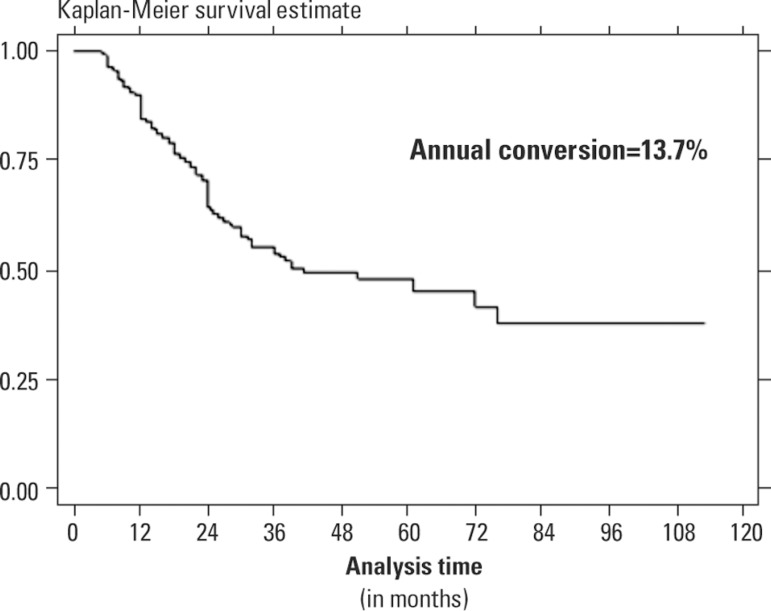
Age-adjusted Kaplan-Meier survival curve for patients with MCI.

Figure 1 shows age-adjusted Kaplan-Meier plots for conversion to dementia in patients with MCI. The annual rate of conversion was 13.7%.

Table 2 provides data on rates of conversion to dementia and also shows the type of degenerative dementia to which MCI patients converted at month 36.

**Table 2:** Conversion from MCI to dementia.

Patients converted (n, %)	83 (34.7)
Cumulative time at risk (months)	6902.16
Incidence rate per month (%)	1.2
Percent converted at month 60 (95%CI)	54.4% (45.5-62.5)
Type of conversion at month 36	
No conversion, n (%)	156 (65.3)
Alzheimer's disease, n (%)	65 (27.2)
Frontotemporal dementia, n (%)	15 (6.3)
Lewy Body Dementia, n (%)	3 (1.3)

As shown in Table 3, converters to dementia were more likely to be older, without occupation at retirement, lower educated and with lower MMSE scores than non converters. At baseline, MCI converters to dementia showed poorer episodic memory (delayed recall and recognition) and semantic memory (naming-BNT, semantic fluency and vocabulary) than MCI non-converters. The presence of intrusions and perseverations was significantly higher in converters. Both populations had similar affective symptoms.

**Table 3:** Summary of characteristics for converters and non-converters.

	Non converters (n=177)	Converters (n=75)	Hazard Ratio	(95% Conf. Int.)		p value
**Sociodemographics**						
Age, years (mean,SD)	70.8 (+7.9)	75.0 (7.0)	1.06	1.03	1.10	<0.001
Sex, male (n, %)	78 (44%)	25 (33%)	0.67	0.41	1.09	0.11
Married (n, %)	121 (68%)	48 (64%)	0.82	0.37	1.82	0.63
Not working (n, %)	127 (72%)	66 (88%)	4.48	1.09	18.3	0.03
Education, median	12	11	0.48	0.30	0.76	0.002
**Neuropsychological assessment**						
Mini Mental State (mean, SD)	27.4 (0.18)	26.1 (0.5)	-4.94	0.69	0.85	<0.001
Memory Battery (Signoret)						
Paragraph recall, median	5.5	4.5	-2.01	0.81	0.99	0.04
Paragraph delayed recall, median	4.5	3.5	-2.51	0.81	0.97	0.01
Verbal Serial learning, median	7	7	-1.37	0.81	1.03	0.17
Verbal Serial free recall, median	5	4	-2.16	0.82	0.99	0.03
Cued recall, median	7	7	-0.04	0.91	1.09	0.96
Recognition, median	11	10	-3.62	0.73	0.911	<0.001
Intrusions, median	0	1	2.67	1.09	1.85	<0.001
Language						
Boston naming test, median	53	48	-4.46	0.90	0.96	<0.001
Semantic fluency, median	16	13	-3.26	0.87	0.96	<0.001
Phonologic fluency, median	12	13.5	0.77	0.96	1.07	0.44
Attention and Executive Functions						
Digit Span, median	8	6	-3.68	0.74	0.91	<0.001
Trail making A, median	55	59	1.30	0.99	1.01	0.19
Trail making B, median	55	59	1.32	0.99	1.00	0.18
Perseverations, mean, SD	50 (28.2)	45 (60.0)	3.06	1.12	1.68	<0.001
WASI						
Vocabulary, median	44	51.5	4.61	1.01	1.03	<0.001
Similarities, median	39	38	2.48	1.00	1.02	0.01
Block design, median	34	34	0.89	0.99	1.01	0.37
Matrix reasoning, median	37	39	1.54	0.99	1.02	0.12
Verbal IQ, mean (SD)	110	99	-4.67	0.94	0.97	<0.001
Performance IQ, mean (SD)	97	91	-3.65	0.94	0.98	<0.001
Global IQ, mean (SD)	103	93.5	-5.05	0.93	0.96	<0.001
**Affective symptoms**						
Beck Depression Inventory, median	9	9.5	0.92	0.98	1.05	0.36

The relative risks for the probability of progression to dementia are listed in Table 4. The multivariate analysis showed that the risk for developing dementia in people diagnosed with MCI increases by 63% in those aged over 75, by 64% with education level less than 12 years, increases two-fold when lacking an occupation, 96% with global IQ less than 111, 93% with a naming score less than 51 on the Boston naming test, and 194% with a score less than 27 on the MMSE. Each additional point on the Global IQ provided a 3.6% increase in protection against the development of Dementia.

**Table 4:** Relative risk predictors for conversion to dementia in multivariate analysis.

Predictor	Hazard ratio	z	p value	95% CI
Age over 75	1.634	2.03	0.043	1.016-2.628
Education less than 12 years	1.640	1.99	0.042	1.075-2.760
Not working	2.409	2.30	0.022	1.137-5.104
Global IQ less than 111	0.964	-2.57	0.010	0.938-0.991
Vocabulary score	3.943	4.42	0.000	2.146-7.237
Naming score less than 51	1.932	2.15	0.032	1.059-3.526
Mini Mental State less than 27	2.947	3.35	0.001	1.566-5.548
MCI amnesic type	2.696	2.44	0.015	1.215-5.977

We performed a factorial analysis (Table 5) in which education (less than 12 years), MMSE (less than 27) and naming (less than 51 on BNT) were used as factor 1 and these accounted for 26.2% of the variability of conversion to dementia, factor 2 was age and lack of occupation at retirement, explaining an additional 15%, factor 3 was vocabulary and presence of intrusions in episodic memory (free recall) explaining an additional 14.3%. Taken together, all these factors accounted for 56% of the variability of conversion from MCI to dementia.

**Table 5:** Factorial analysis.

**(main component factors; 3 factors retained)**				
**Factor**	**Eigenvalue**	**Difference**	**Proportion**	**Cumulative**
1	2.36572	0.93327	0.2629	0.2629
2	1.43245	0.14234	0.1592	0.4220
3	1.29011	0.14252	0.1433	0.5654
**Rotated factor** **Variable **	**Loading**			
		**1**	**2**	**3**
Age		-0.09113	0.76348	-0.10332
Education less than 12		-0.66230	-0.19017	-0.08836
Not Working		0.20192	0.78293	0.07879
MMSE less than 27		0.76879	-0.26735	0.07529
Naming less than 51		0.69490	0.17108	-0.37709
Vocabulary less than 49		0.15144	-0.25318	-0.72847
Intrusions in Memory		0.03003	-0.17461	0.80564
Global IQ less than 111		0.78821	0.14386	-0.07444

## Discussion

There is a clinical cognitive continuum which runs from normal aging to degenerative dementia. Cognitive decline without dementia has commonly been considered a normal consequence of brain aging, but can also indicate the onset of dementia. The boundary between normal aging and very early dementia is becoming a major focus of research. The idea of aging-effects versus disease is not new; in 1962, Kral et al.^24^ described “benign senescent forgetfulness” (BSF) in which fairly unimportant details of an experience (e.g. a name, a place or a date) are not recalled but do not interfere with activities of daily living and do not progress to dementia. Kral also recognized that “differentiation of the benign and malignant types of senescent forgetfulness does not necessarily mean that there are two neuropathological processes”. These diagnostic criteria were not precise, nor were they validated in controlled longitudinal studies. These cognitive changes in aging have been assigned various terms, such as age-associated memory impairment,^25^ late-life forgetfulness^26^ and aging-associated cognitive decline.^27^ These terms have been used largely to explain the limits of normal aging, to characterize individuals who are neither normal nor demented. Such terms were criticized for being inaccurate.^28^

Mild cognitive impairment was first described in the late 1990s by Flicker et al.^29^ and later by Petersen et al.^9^ Petersen proposed a clinical continuum ranging from normal aging through to mild cognitive impairment and on to dementia. MCI was not normal aging: this construct was intended to be a clinical description of persons who were destined to develop dementia.^9^ Currently, an understanding of prodromal states or early clinical presentations of Alzheimer’s disease (AD), is a significant priority since it would aid in early detection, facilitate early treatment, and lead to prevention.^28^

In clinical-based studies the typical rate at which MCI patients’ progress to dementia is 10 to 15% per year. In contrast, the incidence rates for the development of dementia in normal elderly subjects is 1 to 2% per year.^9^ In our clinical referral study involving 239 patients from South America, the annual rate of conversion from MCI to dementia was 13.7%. Among the MCI patients who converted to dementia, 78.3% were AD, 18% FTD, and 3.6% LBD. AD is the natural evolution of MCI which has converted to degenerative dementia.

Several predictive features of conversion from MCI to dementia are beginning to emerge when baseline factors are studied separately. High risk was found for increasing age, lack of occupation in the elderly, low formal education level, and difficulty coping with common situations. At the pre-dementia stage, converted patients showed lower general cognitive function, and greater episodic memory impairment (lower delayed recall with no improvement in recognition and presence of intrusion), semantic memory impairment (naming, verbal fluency and vocabulary), and dysexecutive syndrome (perseveration) than non-converted patients. This amnesic syndrome of the hippocampal type found in prodromal AD (lower delayed recall with no improvement in recognition and presence of intrusion) resemble our findings and was described as pre-dementia stage of Alzheimer’s disease by several groups.^30-33^

In the factorial analysis, education (less than 12 years), MMSE (less than 27) and naming (less than 51) accounted for 26.2% of the variability of conversion to AD, while aging and “leisure inactivity” explained an additional 15%, and vocabulary (less than 49) and the presence of intrusions in memory test explained a further 14.3%. Most of these risk factors were related to the concept of cognitive reserve.^1-7^ Cognitive reserve is the ability to optimize performance through differential recruitment of brain networks, which may reflect the use of alternative cognitive strategies.^1^ Cognitive reserve is the hypothesized capacity of the mature adult brain to resist the effects of disease or injury which are capable of causing clinical dementia in an individual possessing less cognitive reserve^34^. Stern proposed that active and passive components were involved.^1^ Active components encompass high level of education and complex occupations^1^ whereas passive components comprise brain structures involved in memory retrieval, problem solving, and intelligence quotient^34^. Low education level in this population increased the risk of progression to dementia in line with results reported by Kryscio et al.^35^ Epidemiological studies have established low educational attainment as a significant risk factor for dementia.^36-38^

In our study, education level and occupational complexity can be divided into late age as the active factor, and global IQ and old age as the passive factor. All these cognitive reserve-related factors accounted for 56% of the variability of conversion from MCI to dementia.

The main conclusions of this study are:

1. In the MCI “CEMIC Cohort” (239 MCI subjects with 5 years of follow-up), 34.7% converted to Degenerative Dementia within 3 years.
2. Most of the MCI patients converted to Alzheimer Disease (78.3%)
3. The most significant Risk Factors for Conversion from MCI to Dementia were related to cognitive reserve (passive: IQ and age; active: Education and Occupation).

Finally, our results suggest that devising a (cognitive reserve related) risk factor protocol may be helpful in protecting MCI individuals at high risk of conversion. This study can contribute important evidence to guide the decision-making process in routine clinical activity and Public Health policy on aging.
